# Fluorescent Tagged Vaccinia Virus Genome Allows Rapid and Efficient Measurement of Oncolytic Potential and Discovery of Oncolytic Modulators

**DOI:** 10.3390/biomedicines8120543

**Published:** 2020-11-26

**Authors:** Franck Gallardo, Doris Schmitt, Renée Brandely, Catherine Brua, Nathalie Silvestre, Annie Findeli, Johann Foloppe, Sokunthea Top, Sandrine Kappler-Gratias, Charlotte Quentin-Froignant, Renaud Morin, Jean-Michel Lagarde, Kerstin Bystricky, Stéphane Bertagnoli, Philippe Erbs

**Affiliations:** 1 NeoVirTech SAS, 31106 Toulouse, France; sokuntheatop@neovirtech.com (S.T.); skappler@neovirtech.com (S.K.-G.); qfroignant@neovirtech.com (C.Q.-F.); 2 Transgene SA, 67405 Illkirch-Graffenstaden, France; doris.schmitt@transgene.fr (D.S.); renee.brandely@transgene.fr (R.B.); brua@transgene.fr (C.B.); silvestre@transgene.fr (N.S.); findeli@transgene.fr (A.F.); foloppe@transgene.fr (J.F.); 3 Imactiv-3D SAS, 31106 Toulouse, France; renaud.morin@imactiv-3d.com (R.M.); jean.michel.lagarde@imactiv-3d.com (J.-M.L.); 4 Centre de Biologie Intégrative (CBI), Laboratoire de Biologie Moléculaire Eucaryote (LBME), University of Toulouse, UPS, CNRS, 31400 Toulouse, France; kerstin@biotoul.fr; 5 Institut Universitaire de France (IUF), 75231 Paris, France; 6 IHAP, Université de Toulouse, INRA, ENVT, 31076 Toulouse, France; stephane.bertagnoli@envt.fr

**Keywords:** oncolytic vaccinia virus, poxvirus modulators, fluorescence labeling, live cell imaging

## Abstract

As a live biologic agent, oncolytic vaccinia virus has the ability to target and selectively amplify at tumor sites. We have previously reported that deletion of thymidine kinase and ribonucleotide reductase genes in vaccinia virus can increase the safety and efficacy of the virus. Here, to allow direct visualization of the viral genome in living cells, we incorporated the ANCH target sequence and the OR3-Santaka gene in the double-deleted vaccinia virus. Infection of human tumor cells with ANCHOR3-tagged vaccinia virus enables visualization and quantification of viral genome dynamics in living cells. The results show that the ANCHOR technology permits the measurement of the oncolytic potential of the double deleted vaccinia virus. Quantitative analysis of infection kinetics and of viral DNA replication allow rapid and efficient identification of inhibitors and activators of oncolytic activity. Our results highlight the potential application of the ANCHOR technology to track vaccinia virus and virtually any kind of poxvirus in living cells.

## 1. Introduction

Oncolytic virotherapy has recently been recognized as a promising therapeutic approach for cancer treatment. Oncolytic viruses used in cancer virotherapy include adenovirus, herpes simplex virus, reovirus, parvovirus, measles virus and Newcastle disease virus [1]. Compared with other oncolytic viruses, vaccinia virus (VACV) has many unique advantages as a therapeutic vector: it has a high transduction efficiency and a rapid replication cycle, producing mature progeny in just 6 h [2]. It is very safe, replication of VACV is exclusively cytoplasmic, thus eliminating the risk of chromosomal integration [3]. It has a broad-spectrum infectivity and tumor tropism, VACV can infect almost all types of tumor cells [4]. It has a large genome that can accept large foreign DNA inserts of up to 40-kb without affecting infectivity and genetic stability [5]. It has been shown to be capable of evading the immune system enabling more effective systemic delivery [6]. It was widely used in the World Health Organization’s smallpox eradication campaign [7] and after years of large-scale vaccination, the incidence of severe adverse effects was found to be extremely low. Pexa-Vec (JX-594), the most advanced VACV oncolytic product, was derived from a VACV strain engineered to express granulocyte-macrophage colony-stimulating factor (GM-CSF) [8] and has successfully entered Phase III clinical trials.

Like Pexa-Vec, most of the oncolytic VACVs reported to date harbor mutations that inactivate the *J2R* gene. *J2R* encodes the viral thymidine kinase (TK), one of the key enzymes for the synthesis of vaccinia virus DNA. Cellular TK expression is generally decreased in normal cells but increased in rapidly proliferating tumor cells [9]. The TK-deleted VACV requires thymidine triphosphate to ensure its own DNA synthesis from the nucleotide pool present in dividing cells. Deletion of the viral TK gene (*J2R*) maintains replication in tumors while displaying a reduced ability to replicate in other tissues [10,11,12].

Recently, to enhance the safety and efficacy of the oncolytic VACV, we have developed a new oncolytic VACV with targeted deletions of both the *J2R* gene and the *I4L* gene that encodes the large subunit of the ribonucleotide reductase (RR) [13]. We show that the replication of this second-generation oncolytic VACV is highly dependent on cellular RR levels. The double deleted vaccinia virus (∆*J2R*∆*I4L* VACV) is highly attenuated in normal cells yet it displays tumor-selective replication and cell killing, due to the fact that cancer cells express elevated RR levels. ∆*J2R*∆*I4L* VACV is less pathogenic than the single TK-deleted VACV (∆*J2R* VACV) and significant in vivo antitumor effects were observed in multiple tumor models after systemic injection of ∆*J2R*∆*I4L* VACV [13]. TG6002, a double deleted VACV expressing the suicide gene *FCU1* [13], has recently entered into clinical development (NCT03724071, NCT04194034).

Titering of cells is the gold standard to determine viral quantity but lacks information on spreading. Improved techniques for tracking the replication of the virus in vitro are needed. The ANCHOR system is a new DNA labeling technology designed to visualize DNA in living cells. This technique has been used to analyze a variety of processes, including DNA resection upon double-strand break (DSB) induction and chromatin confinement during transcription activation [14,15]. Used in virology, this system has allowed for the first time to investigate the complete viral replication cycle, from infection to cell lysis, of human cytomegalovirus and adenovirus type 5 directly in living cells [16,17]. It has also been used recently to investigate HIV-1 biology [18]. This technology has recently been adapted to tracking baculovirus in living Sf9 insect cells or in whole insect larvae [19]. However, the ANCHOR system has not been applied to cytoplasmic replicating viruses.

To examine whether the system could be used for tracking VACV in living cells, we generated a double deleted ∆*J2R*∆*I4L* VACV containing the ANCH3 target sequence and the gene encoding OR3-Santaka protein. We show that the expression of the ANCHOR system does not detrimentally affect VACV replication. Direct visualization and quantification of viral genomes in the cytoplasm of living cells, confirmed that compared to tumor cells, replication of the double-deleted vaccinia virus is attenuated in normal primary cells. In addition, we used ANCHOR-tagged VACV to screen for molecules that modulate the infection or replication.

## 2. Materials and Methods

### 2.1. Cells

The rabbit kidney epithelial RK13 cells (ATCC CCL37), the human fetal lung fibroblast cell line MRC-5 (ATCC CCL-171) and the human cervical cancer cell line HeLa (ATCC CCL-2) were maintained in Dulbecco’s modified Eagle’s medium supplemented with 10% fetal calf serum (FCS). The human colon cancer cell line HCT 116 (ATCC CCL-247) and the human hepatocarcinoma cell line Hep G2 (ATCC HB-8065) were maintained in Eagle’s minimum essential medium supplemented with 10% FCS. Fresh human hepatocytes were purchased from Biopredic International and maintained in the hepatocyte medium (Biopredic International, Rennes, France). Primary chicken embryo fibroblasts (CEFs) were used for recombination, amplification and titration of viral vectors. CEF cells were prepared as previously described [20] and maintained in MBE supplemented with 5% FCS.

### 2.2. Chemicals and Antibodies

Unless stated otherwise, all chemicals were obtained from Prestwick Chemical. The antibodies used in the current study include mouse antilamin monoclonal antibody (sc-7292; Santa Cruz Biotechnologies, Heidelberg, Germany), mouse antitubulin monoclonal antibody (T5168; Sigma-Aldrich, Saint-Quentin Fallavier, France) and mouse antivimentin monoclonal antibody (M0725; Dako, Glostrup, Denmark). The far red-fluorescent lectin GS-II Alexa Fluor^®^ 647 conjugate was obtained from Thermofisher Scientific (Darmstadt, Germany).

### 2.3. Viruses and Plasmids

All recombinant VACVs were derived from the Copenhagen strain. The ANCHOR3-tagged VACV oncolytic vector (∆*J2R*∆*I4L* ANCHOR3-Santaka VACV, named VACV ANCHOR hereafter) was generated by site-specific recombination in a 2-step process. In the first step, consisting of the insertion of the ANCH sequence into the *I4L* locus, we used the pΔI4L shuttle plasmid containing the ANCH sequence and the selection cassette GFP/GPT, a fusion of the gene encoding the green fluorescent protein (GFP) and the gene encoding guanine phosphoribosyltransferase (GPT), surrounded by portions of the vaccinia *I4L* gene. In this construction, the selection marker GFP/GPT is placed between two homologous sequences in the same orientation. Recombination of the pΔI4L shuttle plasmid was performed into a Copenhagen VACV resulting in the insertion of the ANCH and the GFP/GPT sequences into the *I4L* locus of VACV. Recombinant VACV expressing GPT and that displayed GFP-fluorescence plaques was isolated in a selective medium containing hypoxanthine (15 µg/mL), xanthine (250 µg/mL) and mycophenolic acid (250 µg/mL). The selection marker GFP/GPT was easily eliminated by several passages without selection allowing the growth of gpt^-^ recombinant VACV obtained after intragenic homologous recombination between the two sequences flanking the GFP/GPT cassette. In the second step, the same methods were used to generate the double deleted VACV encoding ANCH and OR3-Santaka (∆*J2R*∆*I4L* ANCHOR3-Santaka VACV) by homologous recombination between the shuttle plasmid pΔJ2R, expressing the OR3-Santaka fusion gene under the control of the p11K7.5 promoter, and the recombinant VACV obtained in the first step (*I4L*-deleted VACV encoding ANCH sequence). Insertion of the ANCH and OR3-Santaka sequences in the recombinant VACV was confirmed by multiple PCRs. VVTG18306 is a double deleted ∆*J2R*∆*I4L* VACV expressing mCherry under the control of the p11K7.5 promoter. Recombinant VACVs were amplified in CEF, purified and virus stocks were titrated on CEF by plaque assay. LifeAct-GFP plasmid, used for visualization of F-actin in living cells, was obtained from Ibidi.

### 2.4. Visualization and Quantification of VACV ANCHOR

VACV ANCHOR was visualized and quantified by high content fluorescence microscopy. Permissive cells (1 × 10^4^ cells per well in 90 µL DMEM) were seeded in a 96-wells tissue culture plate (Corning Cell Bind Black Bottom; Sigma). Twenty-four hours post seeding, cells were infected with 10 µL of a 10-fold serial dilution from virus stock in duplicate. Infected cells were incubated at 37 °C with a 5% CO_2_ atmosphere. One day post infection, cells were fixed with 100 µL formalin for 10 min and nuclei were stained with 100 µL of PBS Hoescht 33342 (1/1000). Infection rate (number of fluorescent cells versus total number of cell) was used to calculate the fluorescence forming unit (ffu/mL).

### 2.5. High-Content Screening Protocol

HCT 116 (1 × 10^4^ cells per well in 189 µL culture medium) were seeded in a 96-wells tissue culture plate (Corning Cell Bind Black Bottom; Sigma). The whole Prestwick Chemical FDA approved library containing 1280 compounds was used at the first round of screening. Twenty-four hours post seeding, cells were treated on a Beckman NxP pipetting robot with 1 µL of library (freshly premade daughter library stock at 0.2 mM) to reach a final concentration of 1 µM. Immediately after treatment, cells were infected at a multiplicity of infection (MOI) of 0.25. Three days post-infection, cells were fixed with 10% formalin for 10 min and nuclei were stained with 100 µL of PBS Hoechst 33342 (1/1000). Automated fluorescence measurements by high-content imaging (HCI) were performed using an Arrayscan VTI HCS microscope and a custom compartmental analysis protocol including a spot detector.

### 2.6. Cell Imaging and Immunofluorescence Staining

For immunofluorescence staining, protocol was used as described [16]. Briefly, cells were fixed for 10 min with 10% formalin (Sigma) and permeabilized with PBS 0.5% TritonX100 for 5 min. Cells were then incubated with PBS BSA1% for 30 min. Primary antibody was diluted in PBS BSA1% and cells were incubated with primary antibodies 2 h. After a wash with PBS, cells were incubated with a secondary antibody at 1/1000 for 45 min. After a wash of PBS, cells were incubated with PBS Hoescht 33342 (1/1000) for 5 min and finally washed with PBS. Cells were imaged directly in PBS. For cell imaging, a Zeiss Axiovert Z1 inverted microscope equipped with a Zeiss 63× (NA1,4) oil immersion objective was used. Z stack, exposure time and image processing were adapted according to the detected signal. For high resolution observation of infection in living cells, replication and cell lysis, cells were seeded in glass bottom 35 mm petri dishes (Ibidi) and images were acquired using a Zeiss Axio Observer Z1, Apotome 2 wide-field fluorescence microscope equipped with live cell chamber at 37 °C with 5% CO_2_. Images were taken as indicated in the figure legend.

### 2.7. Statistical Analysis

Statistical significance was determined by an unpaired two-tailed Student’s *t*-test using GraphPad Prism 5 (GraphPad Software, San Diego, CA, USA). Statistical significance was set at *p* < 0.05.

## 3. Results

### 3.1. Generation of an Autofluorescent VACV

**Figure 1:**
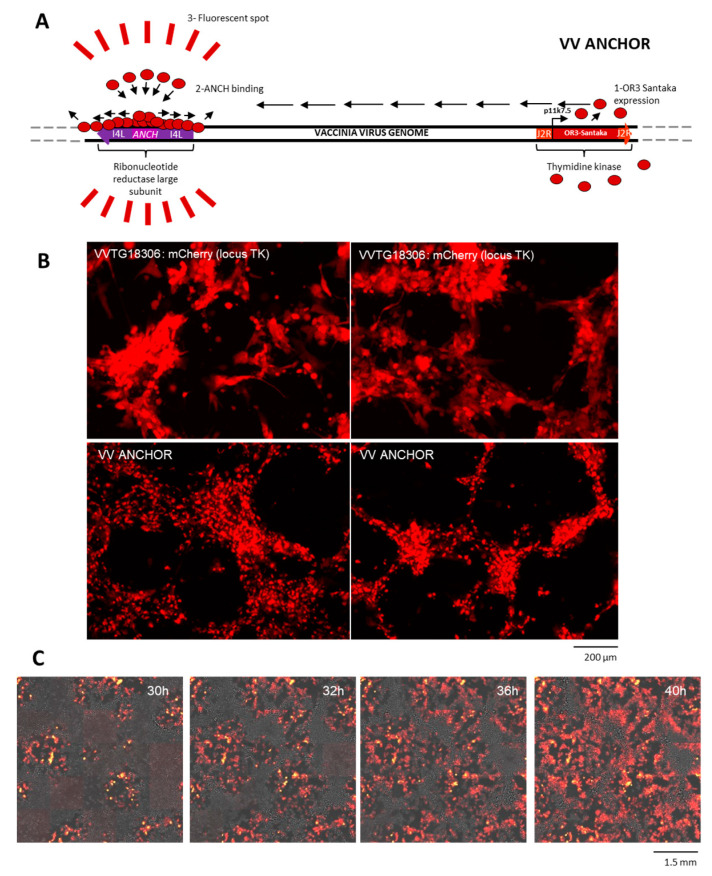
Construction of an autofluorescent ANCHOR tagged vaccinia virus (VACV) that maintains its oncolytic potential. (**A**) Schematic representation of the VACV ANCHOR genome. The ANCH sequence was inserted in the ribonucleotide reductase (RR) locus (*I4L*) by targeted homologous recombination, leading to a VACV *Anch* strain. This strain was then used to insert a p11K7.5 OR3-Santaka cassette at the TK locus (J2R). Upon infection, activation of the p11K7.5 promoter triggers expression of OR3-Santaka (red circles) that recognizes the ANCH sequence, binds and propagates by oligomerization. Accumulation of fluorescent protein onto the tagged viral DNA creates a fluorescent spot that corresponds to the position of the DNA in living cells. (**B**) Fluorescent signal (red) comparison between a classical VACV expressing mCherry (VVTG18036) and the VACV ANCHOR. CEF infected at a multiplicity of infection of 0.1 and 48 h later, the infected cells were observed with an inverted fluorescence microscope Nikon Eclipse Ti (magnification ×100), two fields of view are displayed for each construct. (**C**) HCT 116 cells infected with VACV ANCHOR at a MOI of 0.1 and imaged every two hours under an Arrayscan VTI microscope and showing accumulation of fluorescent spots (red). Reconstruction overlaying brightfield and Santaka channel are displayed over indicated time post-infection.

ANCHOR tagged autofluorescent vaccinia virus (∆*J2R*∆*I4L* ANCHOR3-Santaka VACV) was generated by two rounds of recombination in chicken embryo fibroblasts (CEF). During the first round, the ANCH sequence was inserted into the *I4L* locus of the VACV genome. In the second round, the OR3-Santaka under the control of the p11K7.5 promoter was inserted into the *J2R* locus (Figure 1A). Upon infection, OR-Santaka fluorescent protein is located in the cytoplasm, as this protein does not bear any localization sequence. The protein will bind and oligomerize on any DNA containing the ANCH sequence. Therefore, the protein will accumulate onto the tagged viral DNA. No instability of the sequence over the amplification round was observed and titers produced by the VACV ANCHOR are equivalent to the mCherry VACV construct (VVTG18306). As the OR-Santaka fusion binds the viral DNA, it is possible that Or-Santaka is incorporated into VACV particles during virus production. To determine if this is the case, a 1 µL drop of the VACV ANCHOR crude stock produced in CEF was imaged directly under a fluorescence microscope (Figure S1). The edge of the drop can be easily visualized, as volume decreases at the edge and particles tend to immobilize, fluorescent particles can be perfectly visualized, as opposed to particles in the drop volume that rapidly diffuse due to Brownian motion. Particles of different size can be observed. Since the largest ones likely represent remaining cell debris, the smallest ones may represent viral particles as previously seen for an ANCHOR tagged human cytomegalovirus [16]. Therefore, this result suggests that viral particles are fluorescently tagged even in the supernatant. However, it is possible that this recombinant virus produces a weak signal or displays an altered tropism. To investigate signal strength, we infected CEF at an MOI of 0.1 with VACV ANCHOR compared to a previously designed VACV p11K7.5 mCherry (VVTG18306; Figure 1B). As illustrated in this figure, global fluorescence intensity seems equivalent between both constructs, which is in accordance with the fluorescent protein being expressed under the control of the same promoter in the same locus. The VACV ANCHOR signal seems, however, more punctate than the diffuse mCherry signal. This result shows that the VACV ANCHOR can infect CEF cells and produce a signal at least equivalent to the mCherry construct at low magnification. To investigate if the VACV ANCHOR maintained its oncolytic potential, we infected HCT116 colon carcinoma cell lines at an MOI of 0.1 and imaged infection propagation from 24 h to 40 h post-infection using automated live cell microscopy (Figure 1C, Video S1). Clear fluorescent plaques can be observed at 30 h post-infection. Propagation of fluorescence and cytopathic effect (CPE) can be observed during live cell imaging, indicating that the VACV ANCHOR is able to infect, propagate and kill human cancer cells.

### 3.2. Biological Characterization of VACV ANCHOR

**Figure 2:**
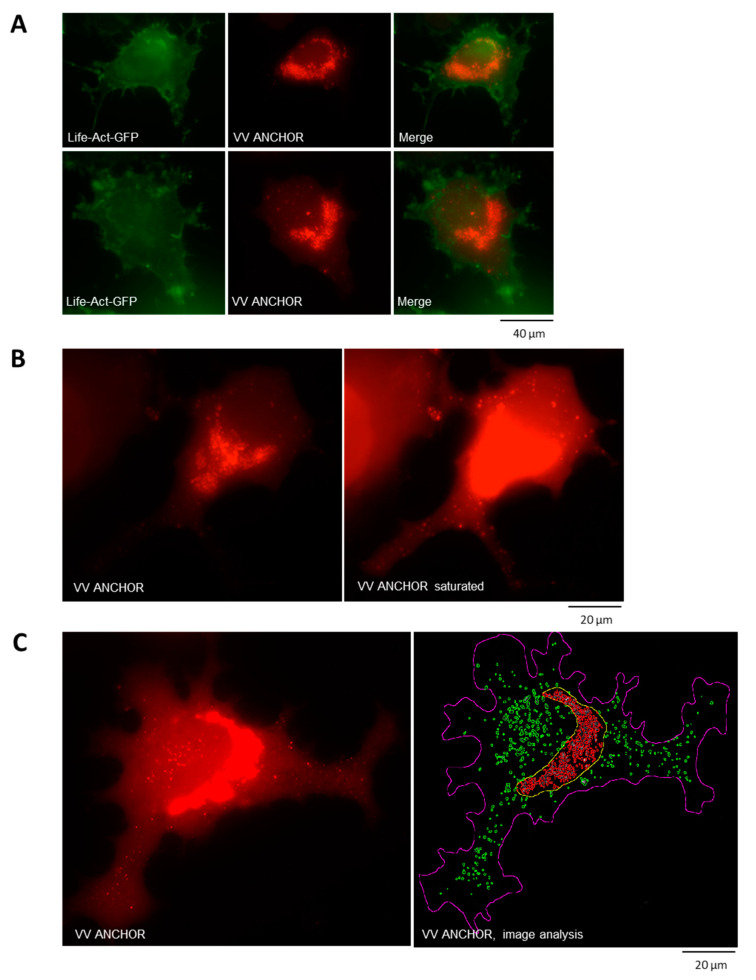
Visualization and quantification of VACV ANCHOR dynamics and copy number in living cells. (**A**) HeLa cells were transfected with plasmid LifeAct-GFP using Extreme Gene HP (Roche). One day post-transfection, cells were infected with VACV ANCHOR at MOI 0.25. Twenty-one hours post-infection, cells were imaged on a Zeiss Axiovert Z1 microscope (GFP lamp 40%, 200 ms exp., Santaka lamp 14%, 100 ms expo). A maximum projection of A 20 frame Z-stack every 1 µm is displayed after out of focus frame removal. Actin is stained in green and VACV DNAs are indicated in red. (**B**) Detection of cytoplasmic particles was done by saturating the fluorescence signal using Fiji (right) from the original image (left) of infected HeLa cells as in A. Images were acquired on a Zeiss Axiovert Z1 63× oil, lamp 40% expo 120 ms, Z stack 20 frames 1 μm interval, Z projection max intensity. VACV DNAs are indicated in red. (**C**) Intracellular quantification of VACV ANCHOR accumulation in living cells was done using Matlab and Image J. Left, VACV DNAs are indicated in red. Right, cell outline is indicated by a purple line, replication center outline is indicated by a yellow line, cytoplasmic VACV DNAs are indicated in green and VACV DNAs in the replication center are indicated in red. For copy number calculation, the smallest detectable fluorescence particles were assigned to a single copy level. According to the integrated intensity of clusters, VACV DNA copy number can be determined reaching 1090 copies in the RC and 592 copies in the cytoplasm.

As described in Figure 1B, the VACV ANCHOR produces a more punctate signal than the mCherry encoded by VVTG18306. To investigate the nature of this punctate signal, we used high resolution imaging of VACV ANCHOR infected HeLa cells transfected with a fluorescent actin staining protein (pLife-Act GFP; Figure 2A) to observe cytoskeleton architecture and dynamics upon infection (Video S2). The punctate fluorescence observed at a low magnification represents a strong accumulation of VACV ANCHOR DNA particles in a crescent shape structure in the center of the cell. This crescent shape structure is often linked to the accumulation of donut-like circular structures that contain several spots of VACV ANCHOR DNA. Additionally, the number and intensity of these fluorescent spots increase over time post-infection (Video S2). The crescent shape structure likely represents the VACV ANCHOR replication center (RC) in living cells. Due to the intensity of the RC signal, small cytoplasmic particles of lower fluorescence intensity are difficult to detect. To see if only the RC can be detected or if single particles in the cytoplasm can be imaged, we oversaturated microscopy pictures, triggering the complete saturation of the RC signal (Figure 2B, left raw data, right saturated). Using this artificial saturation, we can clearly visualize a large number of particles in the cytoplasm of the cell, indicating that particles that are produced in the RC can then be distributed in the cytosol. To investigate the dynamics of these particles, we performed live cell imaging of a cell at 120 ms interval. Saturating the RC signal gives us a clear view of the dynamics of the fluorescent particles (Video S3). To illustrate the dynamics of the VACV ANCHOR in living cells, the time interval was compressed on a single frame using Z-stack Max projection and overlaid with the frame at T0. The resulting images display the initial particle position in yellow and its trajectory in green (Figure S2). As illustrated, large VACV ANCHOR DNA clusters do not display rapid dynamics during the imaging duration (20 frames, 2.5 s, streaming mode). However, the smallest particles display different dynamics. Some particles clearly display diffusive and even oriented movements. However, the vast majority of small particles are almost immobile, indicating that these particles may be bound to subcellular structures. Peak intensity measurements of the smallest particles give a relative intensity value of 49.3 in average (±3.4) whereas particles that display a stronger fluorescence intensity display a value of 98.2 (±17), suggesting that more intense particles contain two copies of the VACV DNA. Altogether this result shows that VACV ANCHOR DNA particle displays a broad range of behavior, from immobile large clusters to highly dynamics particles in the cell. To better quantify the signal provided by the VACV ANCHOR, we used image analysis to determine VACV ANCHOR copies per cell (Figure 2C, left: unprocessed, right: image analysis). The outline of the cell was first estimated using simple thresholding, and then further segmented into RC and cytosolic compartments based on a multivariate Gaussian process assumption. Inside each compartment, corresponding image regions were preprocessed using non-local means denoising, and single VACV clusters were segmented using a hybrid watershed algorithm combining gradient cues and a priori information on particle morphology. The total number of VACV clusters reached 593 in this HeLa cell, comprising 394 clusters in the RC and 199 in the cytoplasm. By making the assumption that the single low intensity diffusive particles that we can observe in the cytoplasm correspond to unique VACV DNA particles, a VACV DNA copy number can be determined, reaching 1682 copies in this cell (1090 in the RC and 592 in the cytoplasm, 2.8 copies/cluster in average in the RC and 2.9 copies/cluster in the cytoplasm). However, we do not know if the number of copies corresponds to fully mature VACV particles or replicating or aborted DNA. Indeed, the presence of the ANCH sequence alone triggers the apparition of fluorescent spots and some of them may reflect uncompleted viral DNA.

**Figure 3:**
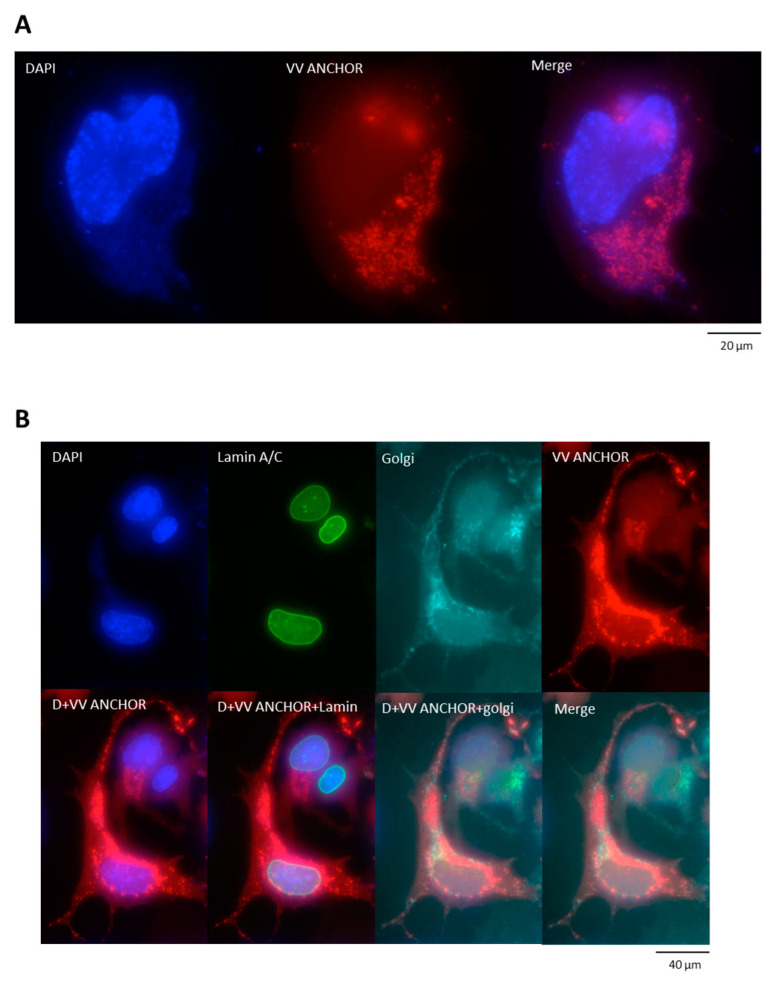

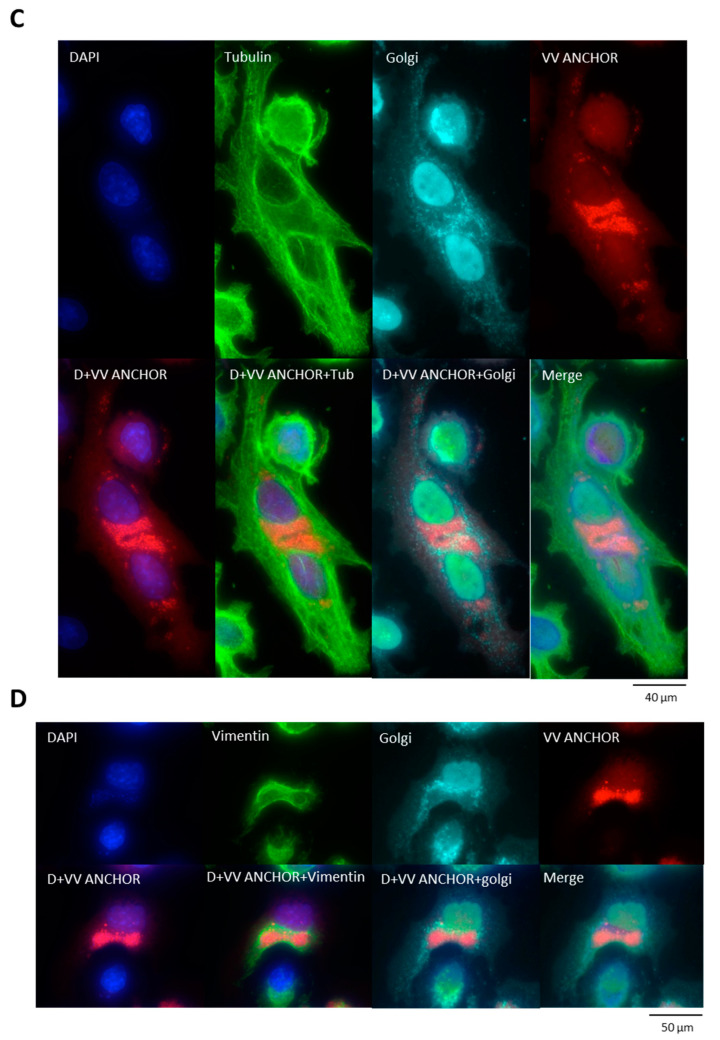
Correlation between VACV ANCHOR infection and the accumulation of markers of interest by immunofluorescence. (**A**) HeLa cells were infected at MOI 1 and fixed with formalin 24 h post-infection. Cells were stained with Hoechst 33342 to visualize DNA. Images are a max projection of A 10 frame Z-stack spaced by 0.5 μm. Nuclear DNA is stained in blue and VACV DNAs are indicated in red. (**B**) As in **A**, but cells were processed for immunofluorescence against lamin A/C. A far-red Lectin GS-II is used to stain the Golgi apparatus. Images are a max projection of A 10 frame Z-stack spaced by 0.5 μm. Nuclear DNA is stained in blue, lamin in green, golgi in light blue and VACV DNAs are indicated in red. (**C**) As in **B**, except an antibody against tubulin (green) was used. (**D**) As in **B**, except an antibody against vimentin (green) was used.

As described in Figure 1 and Figure 2, VACV ANCHOR provides a strong signal that can be used to quantify VACV dynamics and replication. However, we do not know if the signal will be conserved in fixed cells and after processing for other classical staining such as immunofluorescence. To perform this experiment, we infected HeLa cells at a MOI of 0.25 and fixed them 24 h post-infection using formalin (Figure 3A). Cells were then stained with Hoechst 33342. As illustrated, the VACV ANCHOR signal is conserved after fixation. Additionally, the structure of the RC is conserved and appears to be Hoechst positive, confirming the presence of a large amount of DNA in this structure. To go further, we performed immunofluorescence against different cellular structures to verify that VACV ANCHOR signal is maintained and can be correlated to the accumulation of a marker of interest. We used lamin A/C, tubulin and vimentin antibodies, all proteins being involved in cellular architecture (Figure 3B–D and Videos S4–S6, respectively) coupled with the staining of the Golgi apparatus. As shown in Figure 3B, the VACV ANCHOR signal is conserved even after the immunofluorescence protocol, indicating that the signals survive cell permeabilization. Additionally, no defect in the lamin A/C staining was observed, as VACV replication occurs strictly in the cytoplasm. Detection of the microtubule network was also achieved (Figure 3C) and vimentin reorganization upon VACV infection was also detected as structuring the RC (Figure 3D) as described previously [21]. Combined with Z-Stack acquisition and 3D rendering using Imaris software, it allows 3D reconstruction of the cellular volume and the precise determination of VACV position in 3D according to a marker of interest (Videos S4–S6). These results show that VACV ANCHOR provides a strong signal that can be combined with other classical staining methods such as immunofluorescence to correlate VACV infection, localization and replication with cytoskeletal network markers or other cellular or viral elements of interest.

**Figure 4:**
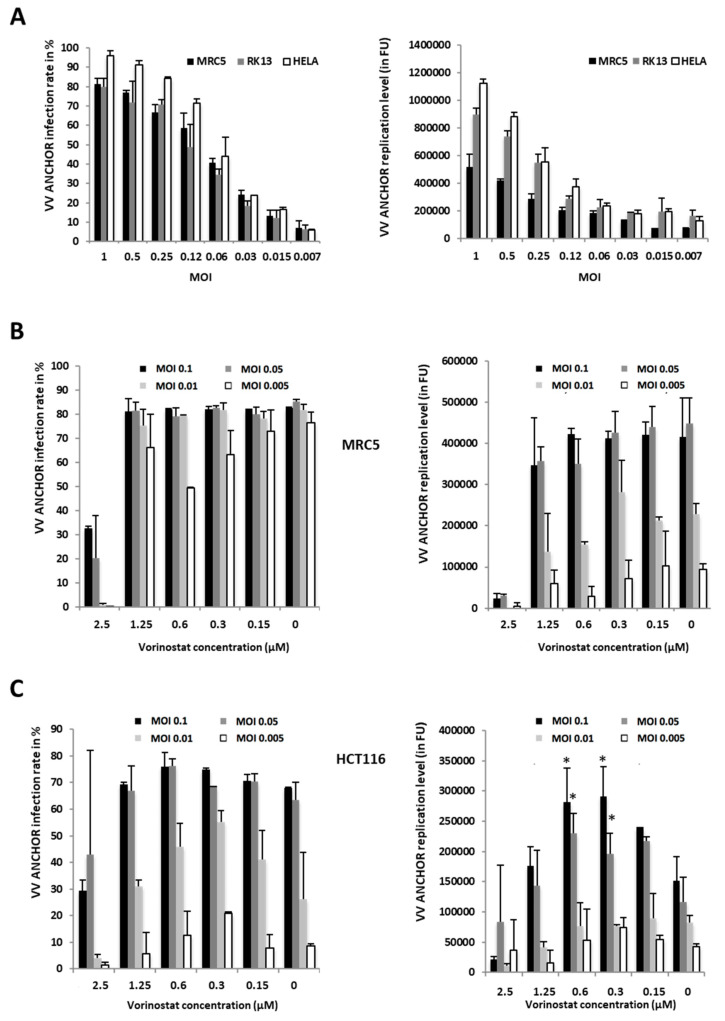
Use of VACV ANCHOR to measure infection capacities and the impact of oncolytic activators. (**A**) Cell lines were plated in 96 well plates and infected with VACV ANCHOR at the indicated MOI. Twenty-four hours post-infection, cells were fixed and stained with Hoechst 33342. Quantification of the infection rate and the VACV ANCHOR replication level was performed on a Cellomics Arrayscan high content microscope. Results are the average of 3 independent wells + SD. (**B**) MRC-5 cells were treated with increasing concentrations of vorinostat and immediately infected with VACV ANCHOR at the corresponding MOI. Cells were fixed and processed for high-content imaging (HCI) as in A, 48 h post-infection. Results are the average of 3 independent wells ± SD. (**C**) HCT 116 cells were treated with increasing concentrations of vorinostat and immediately infected with the corresponding MOI. Cells were fixed and processed for HCI as in A, 48 h post-infection. Results are the average of 3 independent wells ± SD. * *p* < 0.05 compared to cells infected at the same MOI without vorinostat treatment.

As described in Figure 1C, VACV ANCHOR was able to kill colon carcinoma cells, indicating it has conserved its oncolytic properties. To investigate if this virus can be used to rapidly determine oncolytic potential on specific cell lines, VACV ANCHOR infection rate and replication capacities 24 h post infection were determined using several cell lines and different MOIs (Figure 4A). Cells were imaged on a Thermo Cellomics Arrayscan microscope and infection rate (number of Or-Santaka positive cell over the total number of the cell) and replication rate (integrated intensity of VACV ANCHOR fluorescent spots) were determined using a modified compartmental algorithm coupled to a spot detector. At the highest MOI (MOI 1), infection reached almost 100% in the cell line tested, with replication levels exceeding 1 million fluorescence units. When MOI decreased, both infection rate and VACV replication level dropped. HeLa and RK13 cells are the ones that display the best infection and replication rates. VACV ANCHOR can also infect lung fibroblast MRC5 cells and reach a high percentage of infection at high MOI. However, replication rates in this non-cancer cell line are two times lower than in HeLa cells, although this difference is not significant.

### 3.3. Identification of Modulators of Oncolytic Activity

To improve VACV oncolytic activity, it can be necessary to discover oncolytic modulators that increase VACV infection or replication. It has been shown that histone deacetylase inhibitors (HDAC inhibitors) increase replication of VACV specifically in cancer cells [22]. To investigate if VACV ANCHOR responds to HDAC inhibitors, we determined the effect of Vorinostat [23], a HDAC inhibitor used in the clinic, on the infection rate and replication level of VACV ANCHOR in the fibroblast MRC5 cell line and in the cancerous HCT116 cell line (Figure 4B,C). The VACV ANCHOR infects MRC5 cells with high efficiency and the virus efficiently propagates in cell culture, reaching 60–80% infection rate independently of the tested MOI. The replication rate, however, decreased with the MOI. In MRC5 fibroblasts, Vorinostat treatment did not appear to have any effect on VACV ANCHOR replication at all concentrations tested. At the highest concentration, Vorinostat was toxic for the cells. HCT116 colon carcinoma cell lines are also readily infected with VACV ANCHOR and vorinostat did not have any impact on the infection rate. However, it had a significant impact on the virus replication capacity in a concentration dependent manner. Indeed, at high (toxic) and low (no effect) concentrations, Vorinostat did not modify replication capacities. At 0.6 and 0.3 µM and MOI of 0.1 and 0.05, Vorinostat treatment led to a nearly two-fold increase in VACV ANCHOR replication rates. These results underscore the potential of the ANCHOR labeling system to precisely measure the fitness of VACV on different cell lines and to discover compounds modulating infection and replication efficiencies.

**Figure 5:**
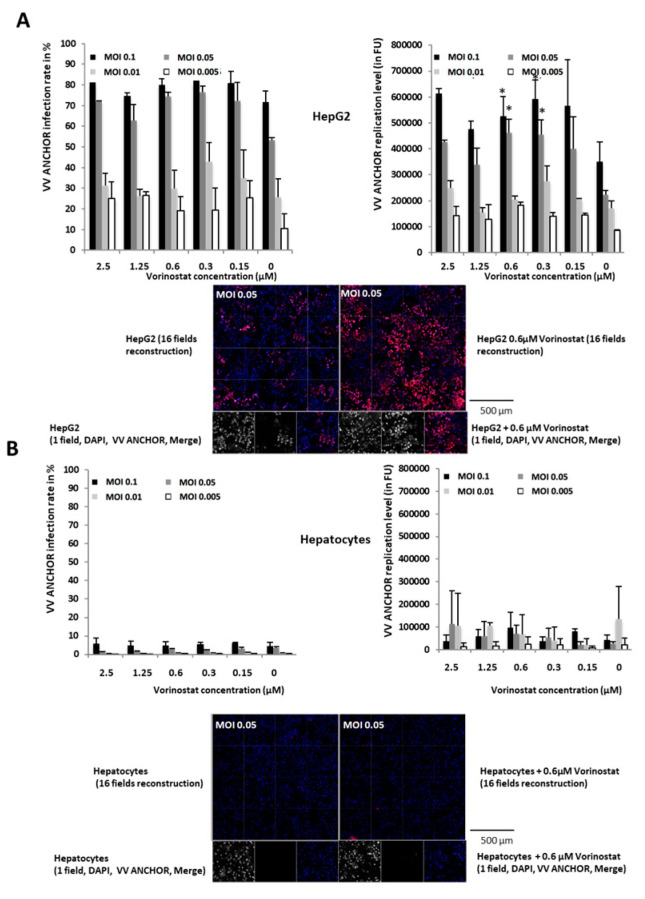
VACV ANCHOR oncolytic potential is restricted to cancerous cells. (**A**) Top: Hep G2 cells were treated with increasing concentrations of vorinostat and immediately infected with the virus at the corresponding MOI. Cells were fixed and processed for high-content imaging (HCI) 48 h post-infection. Results are the average of 3 independent wells ± SD. * *p* < 0.05 compared to cells infected at the same MOI without vorinostat treatment. Bottom: example of Hep G2 cells infected at MOI 0.05 and left untreated (left) or treated with vorinostat at 0.6 µM. Either a field reconstruction of 16 frames or a single frame are presented. (**B**) Top: primary hepatocytes were treated with increasing concentrations of vorinostat and immediately infected with the virus at the corresponding MOI. Cells were fixed and processed for HCI 48 h post-infection. Results are an average of 3 independent wells ± SD. Bottom: example of primary hepatocytes infected at MOI 0.05 and left untreated (left) or treated with vorinostat at 0.6 µM. Either a field reconstruction of 16 frames or a single frame are presented.

As described in Figure 4, VACV ANCHOR can be used to rapidly measure the fitness of infected cell lines. To illustrate its oncolytic potential, we measured infection and replication capacities on human primary hepatocytes versus the Hep G2 liver cancer cell line (Figure 5A,B). In Hep G2 cells, VACV ANCHOR reached 80% infection at the highest MOI 48 h post-infection, and achieved moderate replication rates, reaching 300,000 FU in untreated conditions. As previously described using HCT 116 cells (Figure 4C), vorinostat treatment triggers almost a two-fold increase in VACV DNA replication rate, the best effect being achieved once again at 0.6 and 0.3 µM (Figure 5A, see graph and microscopy pictures at the bottom). Contrary to Hep G2 cells, primary hepatocytes do not divide in culture. According to recommendations of the supplier, cells were plated at 5 × 10^4^ cells per well, contrary to Hep G2 cells that were plated at 1 × 10^4^ cells per well. Therefore, to achieve the described MOI in primary hepatocytes, VACV ANCHOR particle quantity was multiplied by five. Even with this high titer of virus, primary hepatocytes were barely infected, reaching only a low percentage at the highest concentration. In primary cells, as previously described [13], replication of the double deleted VACV is almost completely abolished and is not reactivated by Vorinostat treatment (Figure 5B, see graph and microscopy pictures at the bottom). In absence of viral replication, the presence of small cell debris (false positives) can trigger a jump in the quantification, explaining large error bars. These results show that VACV ANCHOR can efficiently infect and replicate in cancerous liver cells but does not infect and destroy its primary normal cells, indicating that this virus has acquired cancer specific tropism.

**Figure 6:**
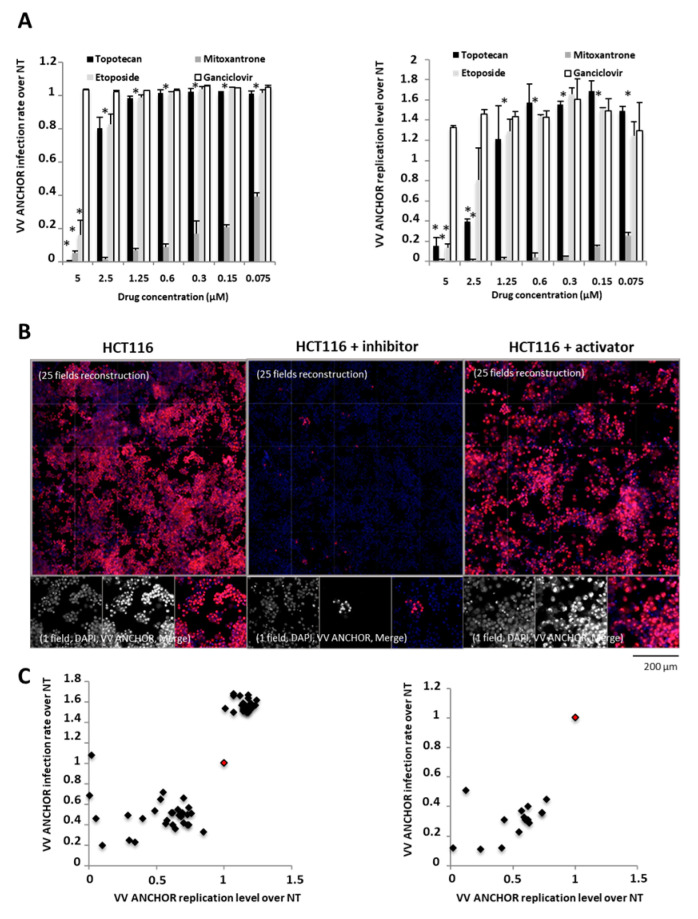
VACV ANCHOR responds to the anticancer treatment and allows fast and efficient discovery of new oncolytic modulators. (**A**) HeLa cells were treated with the indicated concentration of compounds and immediately infected with VACV ANCHOR at an MOI of 0.25. Cells were fixed and processed for HCI 48 h post-infection. Results are expressed as fold increased between treated and non-treated cells (NT). Values are average of 3 independent wells ± SD. * *p* < 0.05 compared to infected cells without drug treatment. (**B**) HCT 116 cell line was used in a high-content screening campaign to identify potential inhibitor or activator of VACV ANCHOR oncolytic capacities. Cells were plated at 1 × 10^5^ cells/well. The day after, cells were treated with 1.25 µM of test compounds and immediately infected with the virus at a MOI of 0.05. Seventy-two hours post-infection, cells were processed for HCI. Example of signal untreated (left), treated with an inhibitor (middle) or with an activator (right) are displayed, either a 25-field reconstruction of the well or a single field at the bottom. (**C**) Scatter plot of the hits identified at the primary (left) and the secondary (right) screening. Value for each compound passing the threshold is displayed (black dot). The control value without drug treatment is indicated by a red dot.

We have shown that VACV ANCHOR can be used to test compounds enhancing replication such as vorinostat. To investigate the behavior of the virus when using known anticancer and antiviral agents, we infected HeLa cells and treated them with several antineoplastic agents (etoposide, mitoxantrone and topotecan) and the antiviral nucleoside analogue ganciclovir, a potent inhibitor of the Herpesvirus family [24] (Figure 6A). Ganciclovir treatment prevented neither infection nor replication of VACV ANCHOR, this treatment being highly specific for herpesviruses infection. Topotecan and etoposide were not able to impact infection but triggered a decrease in VACV ANCHOR replication at concentration ranging from 5 to 2.5 µM. These compounds being specific topoisomerase inhibitors (TopoI and TopoII, respectively) [25], this result confirms that topoisomerase activity is required to reach full replication capacities. The DNA intercalating compound Mitoxantrone, another TopoII inhibitor, is active in dividing and non-dividing cells [26], and was previously described as an antipoxviral agent by blocking processing of viral structural proteins and assembly of mature progeny virions [27]. As described in Figure 6A, Mitoxantrone treatment was able to greatly reduce both the infection rate and DNA replication level of the VACV ANCHOR, even at 75 nM. To discover if other FDA approved compounds can be identified as potent activators or inhibitors of VACV ANCHOR infection and replication, we performed a high content screen using a library of 1280 FDA/EMEA approved compounds (Prestwick Chemicals). We used HCT 116 cells to look for modulator of VACV ANCHOR infection capacities. Primary screening round performed at 1.25 µM and an MOI of 0.05 allowed the identification of several compounds decreasing (Figure 6B, center) or increasing (Figure 6B, right) VACV ANCHOR infection or replication capacities compared to control conditions (Figure 6B, left). Thirty compounds inhibiting at least 40% of infection or replication capacities were identified, and 25 compounds triggered a 50% increase in replication capacity were identified (Figure 6C, left). In a confirmation round, we tested inhibitory compounds at 1 µM for 72 h at MOI 0.05. Of the 30 compounds identified in the first round, 15 of them confirmed a potent inhibitory effect (Figure 6C, right), some of them being able to reduce either replication or infection rate by tenfold. We are investigating the effect of both inhibitors and activators and the results will be published elsewhere.

## 4. Discussion

VACV is unique among most DNA viruses in that its replication occurs entirely in the cytoplasm of the infected host cells [28]. Genome replication and assembly of VACV particles occur in cytoplasmic domains often referred to as replication centers (RCs) or viral factories [29,30,31]. Early after infection, many of the RC are entirely surrounded by membranes of the endoplasmic reticulum, which become dispersed at the start of intermediate or late viral protein synthesis and virus assembly [32]. Here, we adapted the ANCHOR system to the biology of poxvirus. OR-Santaka expression is triggered by the synthetic p11K7.5 early late promoter [20], inducing a rapid and strong expression of this protein that can bind and oligomerize onto the ANCH tagged viral DNA. Recombination of the ANCHOR system in the VACV DNA led to the production of ANCHOR tagged virus without any defect in production or oncolytic capacities. Direct imaging of the produced viral particles indicated that VACV ANCHOR particles are fluorescent even in the supernatant, suggesting that the OR protein is conserved during the poxvirus maturation process, a phenomenon that has already been described for other large viruses such as human cytomegalovirus [16]. High resolution imaging allowed visualization of the VACV ANCHOR replication center and visualization of single particles inside the cytoplasm. Particle quantification and dynamics study demonstrated that the replication center contains numerous almost immobile VACV ANCHOR copies. Cytoplasmic particles display a broad diversity of signals; from single VACV ANCHOR particles displaying active movement to cluster of several copies of VACV DNA adopting confined and slow diffusive motion. Scoring of VACV ANCHOR accumulation led to the measurement of more than a thousand copies per cell. Considering the 190 kb of the VACV genome and the calculated number of VACV ANCHOR DNA copy (1682, Figure 2C), 320 Mb of viral DNA accumulates in infected HeLa cells. This means that upon acute infection, 10% of the total cellular DNA is of a viral nature. Unlike the fluorescent protein tagged viruses described previously [33], this is the first time that viral DNA was specifically and directly observed in live cells. Additionally, we were able to perform single cell determination of the VACV ANCHOR copy number. For the first time, we were also able to correlate VACV ANCHOR replication state to the localization of a marker of interest. By combining VACV ANCHOR with immunofluorescence staining techniques, one can determine the correlation between the VACV replication state and the accumulation and localization of a marker of interest such as tubulin, lamin and vimentin.

Investigation of VACV replication is classically achieved either by qPCR or by plaque assays. qPCR will determine the VACV copy number in a cell population, it is an averaging technique that to date cannot provide precise cell to cell-based accumulation. qPCR will score any kind of VACV copies even if they are not fully mature and therefore non-infectious. Titration by plaque assay is efficient to determine virus titer and only scores the presence of fully infectious particles. It is, however, laborious and time consuming. Only fully infectious particles are scored by the ANCHOR technology, as non-mature particles or free VACV DNA will be unable to enter cells and activate expression of the p11K7.5 promoter. Coupling imaging to the image analysis algorithm, one can measure VACV DNA accumulation in living cells.

We used VACV ANCHOR to rapidly investigate in vitro virus replication on different cell lines, comparison of cancer versus primary cell lines, response to known activators of replication and identification of potent oncolytic modulators. As scoring of compound efficiency is achieved by phenotypic readout, poxvirus activators or repressors are identified during the same experiments. These compounds can be further used to inhibit poxvirus infection in case of outbreak, activate the oncolytic potential of a specific oncolytic construct or inhibit its activity in case of uncontrolled replication in the patient. With the increase of oncolytic vectors going into clinical phases, it will be of importance to find compounds that can increase their potential or control safety parameters after administration in humans. Additionally, our technology can provide valuable data on the interaction between VACV oncolytic activities and pre-existing treatment. Our results indicate that combining mitoxantrone with the virus should be avoided in clinical trials, due to the inhibition of the oncolytic potential of double deleted VACV by this chemotherapy.

All together the design and validation of the ANCHOR technology for poxvirus infection will pave the way for rapid and efficient investigation of poxvirus biology at large directly using live cell microscopy.

## 5. Conclusions

Our results show that the ANCHOR DNA labeling technology provides an efficient tool for the study of oncolytic vaccinia virus.

## Supplementary Materials

Supplementary Materials can be found at https://www.mdpi.com/2227-9059/8/12/543/s1.
